# Eosinophilic esophagitis in Japanese patients: A mild and slow-progressing disorder

**DOI:** 10.1371/journal.pone.0206621

**Published:** 2018-11-02

**Authors:** Hiroki Sato, Terasu Honma, Yujiro Nozawa, Takashi Owaki, Michitaka Imai, Tomoe Sano, Akito Iwanaga, Keiichi Seki, Toru Ishikawa, Toshiaki Yoshida, Shuji Terai

**Affiliations:** 1 Department of Gastroenterology, Saiseikai Niigata Daini Hospital, Niigata, Japan; 2 Division of Gastroenterology and Hepatology, Niigata University Medical and Dental Hospital, Niigata, Japan; University Hospital Llandough, UNITED KINGDOM

## Abstract

**Background and aim:**

Awareness of eosinophilic esophagitis (EoE) has gradually increased in Japan, therefore the characteristics of this disease in the Japanese patient population need to be elucidated. This study aimed to investigate the features of EoE in the Japanese population.

**Methods:**

During a 2-year period, all gastrointestinal endoscopies were performed with maximum attention being paid to identify EoE through endoscopic findings. Clinical features and findings were analyzed among this population.

**Results:**

Among a total of 8589 patients (general gastrointestinal endoscopy, performed for evaluation of symptoms or disease follow-up: 3669; medical check-up endoscopy, routinely performed in asymptomatic patients: 4920), 17 patients (0.20%) were diagnosed with esophageal eosinophilia (mean age ± standard deviation: 44±11.9 years; 1 female). Only 6 patients with esophageal eosinophilia were diagnosed by general gastrointestinal endoscopy; among them, 3 patients had dysphagia and 3 were asymptomatic. The remaining 11 patients were diagnosed by medical check-up endoscopy. All patients were treated with a proton pump inhibitor (PPI); 5 were diagnosed with EoE and 12 with PPI responsive esophageal eosinophilia. Chronological endoscopy analysis showed that EoE findings could be observed for a mean of 6.1 years prior to diagnosis, and the disease did not significantly progress in severity.

**Conclusions:**

Most Japanese patients with EoE have mild and slowly progressing disease, which can be diagnosed when close attention is paid to the endoscopic findings. Medical check-up endoscopy in Japan could be a great opportunity for the early diagnosis of EoE.

## Introduction

Eosinophilic esophagitis (EoE) is a Th2-allergy disorder in the esophagus mainly caused by food. Chronic eosinophil-dominant inflammation triggers obstruction-related symptoms, such as dysphagia [1, 2]. In EoE, eosinophil infiltrations are localized in the esophagus; therefore, the disorder is clearly different from eosinophilic gastroenteritis (EoGE), in which eosinophil infiltration involves single or multiple segments extending from the stomach to the colon, with or without esophageal involvement [3, 4]. In Western countries, the prevalence of EoE has rapidly increased over the past 2 decades. Currently, EoE represents a significant clinical problem associated with marked deterioration in patients’ quality of life and enormous healthcare cost [5, 6]. In Japan, the first patient with EoE was reported in 2006 [7], and the awareness of this disorder, which may increase in prevalence in the future, is gradually growing. However, reports focusing on the clinical characteristics of EoE in Japanese patients are still few [8, 9].

## Methods

During the 2-year period from April 2016 through March 2018, all gastrointestinal endoscopies (GIE) at our hospital were performed with maximum attention being paid to the major findings of EoE using the validated EoE Endoscopic Reference Score (EREFS): decreased vascularity or edema, esophageal rings, white exudates, linear furrows, and strictures. The total grade of the 5 major EREFS findings was calculated and categorized on a 10 point scale, from 0 (normal esophagus) to 10 (the most severe disease), following Hirano’s EREFS classification [10]. More than 4 biopsies were obtained from both the proximal and distal esophagus to maximize the likelihood of detecting esophageal eosinophilia (EE) in all patients in whom EoE is being considered. Patients with histological confirmation of dense eosinophil infiltration (≥15 eosinophils [eos] per high-power field [hpf]) localized in the esophagus were diagnosed with EE [11]. All were treated with proton pump inhibitor (PPI) and categorized as EoE or a variation of EoE: PPI-responsive EE (PPI-REE) [12]. Two types of GIE were performed: general endoscopy, performed for symptomatic patients, disease follow-up, etc., and covered by public insurance; and medical check-up endoscopy, routinely performed for non-symptomatic patients. Secondary causes of EE, and patients with EoGE were excluded.

Patients diagnosed with EE were given a 2-month course of a PPI followed by endoscopy with biopsies [11]. PPI-REE was defined as cases with complete remission, including clinical (lack of any EoE-attributed symptoms), endoscopic (complete absence of inflammatory signs), and histological remission (<5 eos/hpf) [13]. In the absence of these criteria, they were diagnosed as EoE.

The present study was approved by the Saiseikai Niigata Daini Hospital review board (Review Board No. E16-16) and was carried out in accordance with the Declaration of Helsinki. Written informed consent was obtained from all patients.

### Statistical analysis

Continuous variables are expressed as means ± standard deviations. Non-continuous variables are expressed as percentages of positive findings.

Statistical comparison was performed using the t test and the Mann-Whitney U test for parametric and nonparametric data, respectively, with significance assumed at P <0.05. Statistical analysis was performed using SPSS, Version 20 (IBM SPSS Inc., Chicago, IL).

## Results

### Mild disease severity and good response to PPI therapy

**Fig 1:**
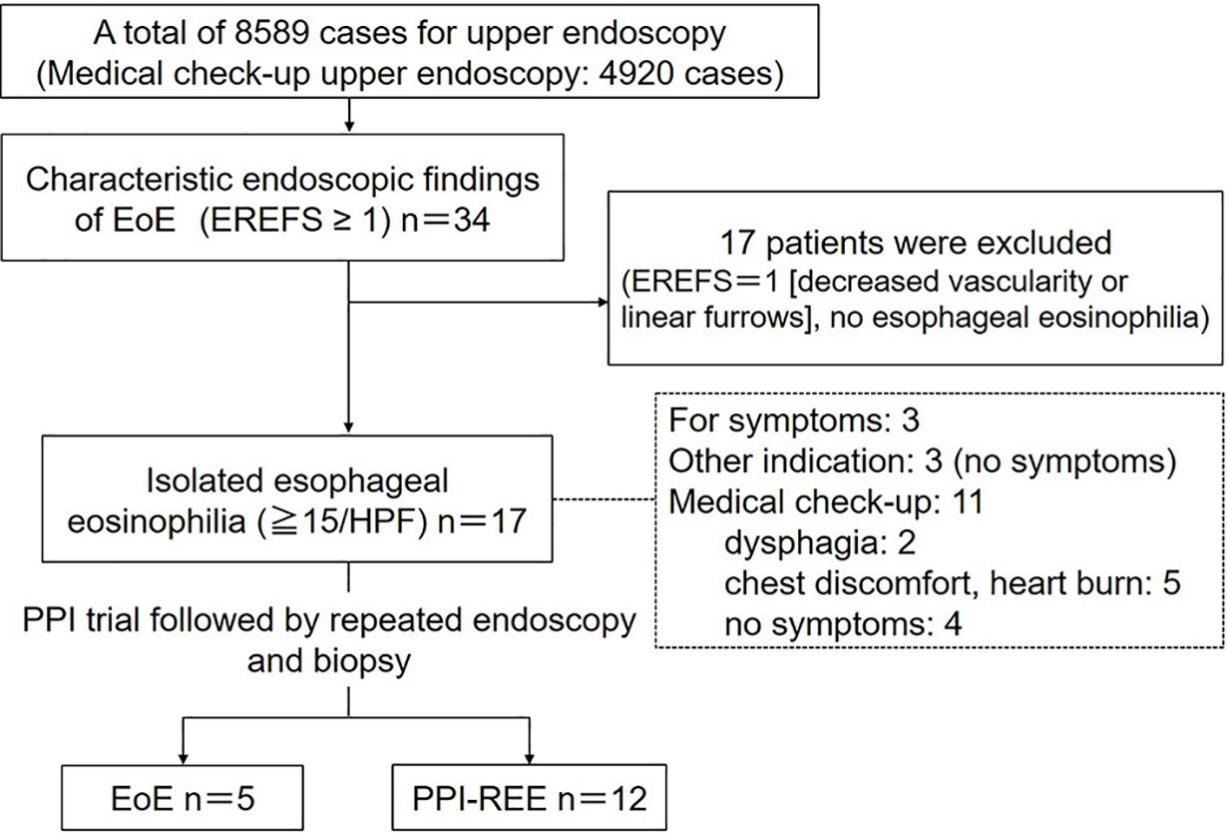
Diagnostic diagram of eosinophilic esophagitis (EoE) in this study. Among a total of 8589 patients who underwent gastrointestinal endoscopy, 17 (0.20%) were diagnosed with esophageal eosinophilia (EE). Six cases of EE were diagnosed by general gastrointestinal endoscopy. Among them, 3 had dysphagia and 3 were asymptomatic. The remaining 11 cases were diagnosed by medical check-up endoscopy routinely performed in asymptomatic adults. On interview, 2 patients had dysphagia, 5 had slight chest discomfort or heart burn, and 4 were asymptomatic. EoE was diagnosed in 5 patients and proton pump inhibitor–responsive esophageal eosinophilia in 12. All were treated by proton pump inhibitor.

In a total of 8589 patients who underwent GIE, 3669 underwent general endoscopy and 4920 underwent medical check-up endoscopy. Seventeen patients (0.20%) were diagnosed with EE (mean age ± standard deviation [SD]: 44 ± 11.9 years; 1 female; maximum ± SD eos/hpf on histology: 66.7 ± 30.5). A history of allergy was identified in 13 patients (asthma: 7; rhinitis: 9; atopic dermatitis: 5; food allergy: 2 [duplicate answers allowed]). On GIE in these 17 patients, edema, furrows, exudates, rings, and strictures were observed in 16, 14, 1, 5, and 3 patients, respectively. Only 6 patients with EE were diagnosed by general gastrointestinal endoscopy. Among them, 3 underwent endoscopy for dysphagia, and 3 were asymptomatic and underwent endoscopy for other indications. The remaining 11 patients were diagnosed by medical check-up endoscopy. Among them, 2 patients reported dysphagia, 5 reported chest discomfort or heartburn, and 4 were asymptomatic. EoE was diagnosed in 5 patients and PPI-REE in 12. All were treated by PPI (Fig 1). The characteristics of patients with EoE and PPI-REE are summarized in Table 1, and the two groups were analyzed for any differences in clinical characteristics.

**Table 1:** Clinical characteristics of patients with EoE and PPI-REE.

	EoE (n = 5)	PPI-REE (n = 12)	P value
**Male/female**	5/0	11/1	NS (0.79)
**Age, mean (±SD), y**	53.4±14.7	47.0±9.8	NS (0.45)
**Atopy**	80.0% (4/5)	75.0% (9/12)	NS (0.87)
**Patients with dysphagia**	40.0% (2/5)	33.3% (4/12)	NS (0.83)
**Serum eosinophil levels (cells/μl)**	472.1±218.3	348.1±256.3	NS (0.38)
**Total IgE (IU/ml)**	708.7±609.0	449.7±1027.5	NS (0.56)
**Endoscopic findings**			
**Edema**	80.0% (4/5)	100% (12/12)	0.53 (NS)
**Fixed rings**	60.0% (3/5)	16.7% (2/12)	0.17 (NS)
**Exudates**	20.0% (1/5)	0% (0/12)	0.53 (NS)
**Furrows**	100% (5/5)	75.0% (9/12)	0.43 (NS)
**Stricture**	20.0% (1/5)	16.7% (2/12)	NS (0.92)
**Histological findings (Eos/HPF)**	76.8±24.4	60.5±30.6	NS (0.32)

Patient characteristics were retrieved from a computer database.

EoE, eosinophilic esophagitis; PPI-REE, proton pump inhibitor-responsive esophageal eosinophilia; NS, not significant; SD, standard deviation; HPF, high power field

### Endoscopic findings did not progress to a severe stage

**Fig 2:**
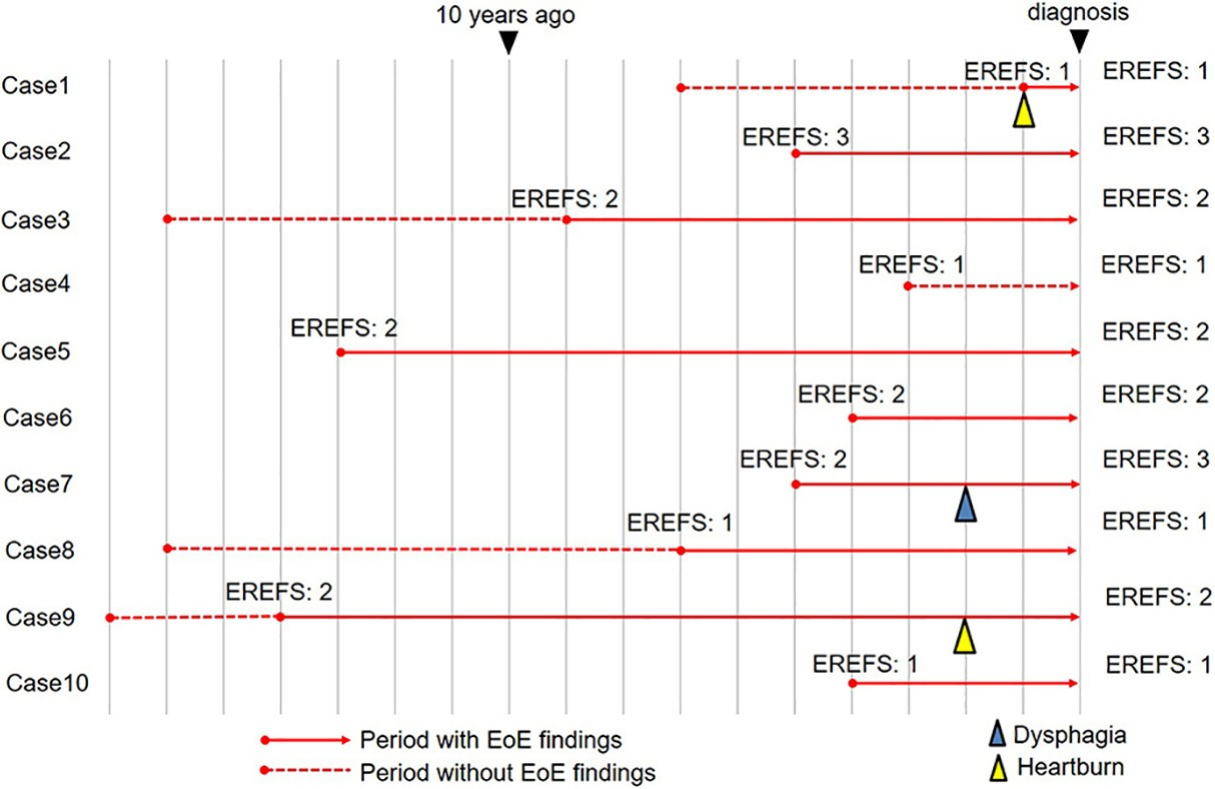
Time series of endoscopic findings using an endoscopy database. The red solid line indicates the period when the endoscopic findings of eosinophilic esophagitis (EoE) were observed, and the dotted line indicates the period when endoscopic findings of EoE were not identified. Over a mean period of 6.1 years during which endoscopic findings of EoE were observed, only 1 patient developed dysphagia (blue triangle indicates the point of dysphagia) and 7 were asymptomatic (yellow triangle indicates the point of chest discomfort).

**Fig 3:**
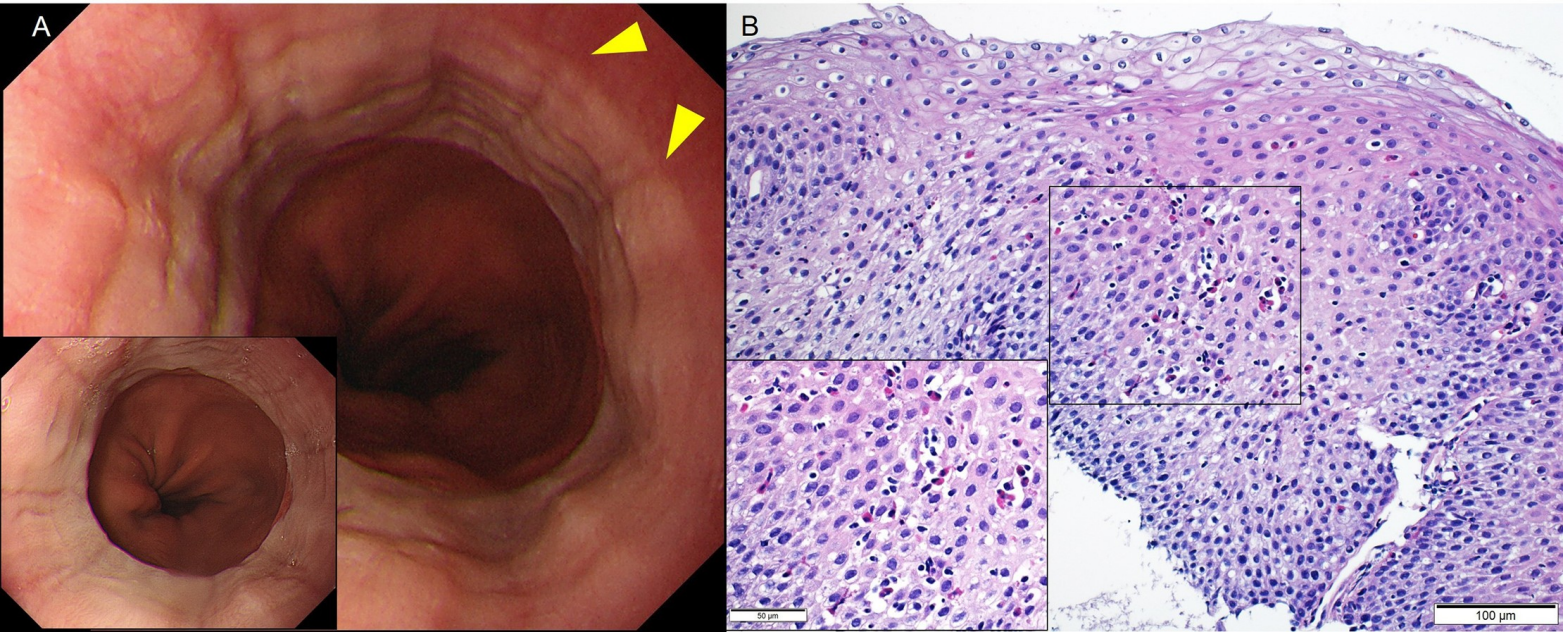
(a) Endoscopy showing loss of vascularity and longitudinal furrows (yellow triangles) in the lower esophagus (Eosinophilic Esophagitis Endoscopic Reference Score [EREFS]: 2); esophageal eosinophilia was diagnosed on histology. The patient was asymptomatic. Inset shows the endoscopy finding 4 years prior in the same patient. Loss of vascularity and longitudinal furrows were already visible (EREFS: 2). (b) Esophageal biopsy demonstrates severe eosinophilic infiltration (200 X). Inset magnification, 400 X.

In 10 patients with EE, the repeated endoscopy findings could be analyzed retrospectively using our endoscopy database, because these patients underwent annual endoscopy before the diagnosis. The latent period between initial endoscopic findings and symptom development was examined (Fig 2). In 9 patients (excluding case 4), endoscopic findings of EoE (red solid line) were observed to begin at a mean of 6.1 years prior to diagnosis (range: 1–14 years). The total EREFS was 2.2 on initial endoscopy, and had not progressed at the time of diagnosis (EREFS of 2.4 on diagnosis). Only 1 patient developed dysphagia during the disease period (case 7), although another 2 patients reported slight chest discomfort (cases 1 and 9). The remaining 7 patients did not develop any symptoms (Fig 3).

## Discussion

This study indicates that in Japan, even mild or asymptomatic EoE can be detected by detailed endoscopic examination, and medical check-up endoscopy could be a good opportunity for the early diagnosis of EoE. In Japan, the local government or employer provide annual medical screening for the inhabitants or employees to promote their health and to detect and treat cancers earlier. In general, those undergoing medical check-up are asymptomatic, middle aged or elderly, and more likely to be male, reflecting the make-up of the Japanese workforce. This represents a patient population susceptible to EoE (middle aged and male predominant). In our series of EoE diagnosed on medical check-up, mildly symptomatic patients who did not require hospital admission, and asymptomatic patients were included. The most appropriate management for these asymptomatic patients remains unclear and needs to be addressed next.

On endoscopy, edema and furrows are considered to be findings highly indicative of EoE. Our results demonstrate that before patients developed dysphagia, EoE showed a year-unit latent period, or in some cases greater than 10 years, during which mild endoscopic inflammatory findings including edema, white exudates, and furrows were identified. As symptoms develop, such endoscopic findings may become more apparent, and fibrotic findings (esophageal rings and stenosis) are observed late in the disease course. However, in our Japanese case series, endoscopic findings did not progress to a severe stage, and only 1 patient developed dysphagia during the observation period. Moreover, our series included more asymptomatic or mildly symptomatic adult patients with EE compared to that in western countries. These results indicate the following: (1) EE in Japanese patients is mild in severity, and (2) only a small portion of patients with EE progress to the severe phase. In this study, pediatric patients with EE were not included, and may be rare in Japan. Therefore, late onset of the disorder may correlate with a mild form in Japan. Moreover, the rate of PPE-REE was higher (70.6%; 12/17) in our series than that in Western countries, which may also be associated with the mild disease severity in the Japanese population. It has been established in recent years that EoE and PPI-REE are indistinguishable clinically [14] and by molecular assays [15]. Thus, distinction between the two is artificial, and PPI-RRE should be considered as EoE [16]. This study indicates that the difference between the two is only due to the diseases’ severity.

There are several limitations to our analysis. First, the analysis of the incidence rate of EoE in our region is difficult in this study, although the prevalence rate of EE was 0.20%, therefore the incidence of EE in Japanese patients is still considered to be low. One of the reason is that medical screening targets the employees, regardless of whether they are inhabitants of the local community. Another reason is that it is impossible to define the background population of our hospital. Second, in our retrospective endoscopy analysis, the number and quality of esophageal pictures were not always adequate; therefore the onset of EE may actually be earlier. Third, not all the histological findings at disease onset were obtained due to the same reason.

In conclusion, EoE in the Japanese population is associated with slow-progressing and mild symptoms. Medical check-up endoscopy represents an opportunity for the early diagnosis, potentially preventing future esophageal stenosis and other severe symptoms.
